# The effect of flavorings on PAHs level in the roasted sunflower seeds

**DOI:** 10.1038/s41598-023-44994-8

**Published:** 2023-10-16

**Authors:** Parisa Shavali-gilani, Najmeh Yazdanfar, Gholamreza Jahed-khaniki, Ebrahim Molaee-aghaee, Parisa Sadighara

**Affiliations:** 1 Division of Food Safety and Hygiene, Department of Environmental Health Engineering, School of Public Health, Tehran University of Medical Sciences, Tehran, Iran; 2 Iranian Research and Development Center for Chemical Industries, ACECR, Tehran, Iran

**Keywords:** Environmental sciences, Diseases, Risk factors

## Abstract

The amount of polycyclic aromatic hydrocarbons (PAHs) can be reduced by food additives. In this study, the impact of various flavors was investigated on the formation of PAHs in roasted sunflower seeds. PAHs was measured in the shell and kernel of sunflower with the flavors of lemon, golpar (hogweed), salt, ketchup and raw sunflower. Measuring the amount of PAHs was analyzed by Gas chromatography–mass spectrometry (GC–MS). PAHs with low molecular weight were detected. The total of PAHs of sunflower seeds were in the range of 0.4–3.2 mg kg^−1^. The lowest amount was related to the hogweed kernel, and the highest amount was related to the lemon. High molecular weight PAHs were not detected because the temperature did not rise above 100 °C during roasting. Some flavors, such as hogweed can reduce the amount of PAHs because of their antioxidant properties. On the contrary, the PAHs level with lemon juice was higher than other flavors.

## Introduction

Polycyclic aromatic hydrocarbons (PAHs) are a category of over one hundred chemicals that are found in organic materials like coal, oil, fossil fuel, wood, garbage, and tobacco during incomplete combustion. Based on the number of benzene rings, they are categorized into two classes: “light PAHs” and “heavy PAHs”, PAHs with 5 or more carbon atoms are in the heavy category, and PAHs with less than 5 benzene rings are in the light category. The heavy PAHs, compared with the light ones, are higher stable, teratogenic and carcinogenic^1–3^. Human exposure to PAHs is through the air, water, and food, but the highest exposure is observed through food^4–6^. Food contaminated with PAHs primarily appears from production practices^7,8^. Unprocessed plant foods may be contaminated with PAHs through contaminated water, air and soil^7^.

Considering the confirmed risk of PAHs to human health, it is very necessary to measure these components in environmental media and food^9^. The International Agency for Research on Cancer (IARC^10^) has classified benzo[a]pyrene as a group 1 carcinogen. While benz[a] anthracene (BaA), chrysene (Cry), benzo[k] fluoranthene (BkF) and benzo[b] fluoranthene (BbF) are classified in group 2B (probably carcinogenic to humans). The European Union (EU) Commission has set various maximum levels for PAHs in certain foodstuffs, focusing on foods containing fats and oils or where the food is smoked. According to these guides, the assumed benzo[a]pyrene levels in coffee beans and products derived from them is 5 µg kg^−1^ fat, and in oils used by humans is 2 µg kg^−1^^11^. Due to the large family of PAHs, several key compounds have been introduced to indicate the presence of PAHs in food. The first PAH proposed as an indicator was Benzo[a]pyrene (BaP). But in 2008, the European Food Safety Authority (EFSA) declared that BaP alone cannot be a good indicator of the occurrence and carcinogenic effect of PAHs in food. So Eight compounds were selected as the most suitable indicators [PAH8; chrysene, benzo[k]fluoranthene, dibenz [a,h] anthracene, benzo[a] anthracene (BaA), BaP, indeno[1,2,3-pyrene (IP), benzo[b]fluoranthene (BbF), and benzo [ghi] perylene (BghiP) (EFSA, 2008).

Roasted sunflower seeds can be a source of PAHs due to roasting and the presence of unsaturated fats^12^. Consumption of these nuts is very popular in Iran. Roasting involves a number of physicochemical changes including dehydration and chemical reactions^13^. Among the food preparation processes, roasting, smoking and grilling more than other processes lead to an increase in the amount of PAHs. Therefore, the source of PAH in roasted sunflower seeds is the contamination of raw sunflower seeds with environmental contaminants and contaminants produced during the roasting process^14^. In several studies, the effect of food additives and the formation of PAHs in grilled meats was investigated. The results of these studies show that the physicochemical properties of food affect the amount of PAH in food products^15–17^. These properties include pH, moisture content, antioxidant activity, fatty acid composition, etc. Different flavorings can affect these properties and thus the production of PAHs.

Considering the relatively high consumption of sunflower seeds in the Iranian basket, suitable solution should be adopted to reduce PAHs in roasted sunflower seeds. The aim of this study was to investigate the effect of different flavors on the amount of PAHs in roasted sunflower seeds.

## Materials and methods

### Materials

Calibration mixture of sixteen PAHs standards dissolved in acetonitrile, were purchased from Supelco (Bellefonte, PA, USA). These PAHs, include naphthalene (Nap), acenaphthylene (Acy), acenaphthene (Ace), fluorene (Fl), phenanthrene (Phe), anthracene (Ant), fluoranthene (Flu), pyrene (Py), benz(a)anthracene (BaA), chrysene (Chr), benzo(b)fluoranthene (BbF), benzo(k)fluoranthene (BkF), benzo(a)pyrene (BaP), indeno (1,2,3-cd) pyrene (IPy), dibenz(a,h)anthracene (DahA), and benzo(ghi)perylene (BghiP). The concentration of each PAHs in the mixture was 2000 mg L^−1^. Working standard solutions were prepared daily with analytical grade Methanol. Other solvents were purchased from Merck (Darmstadt, Germany). All other chemical materials used of the highest purity too.

### Sampling

Samples of sunflower seeds with different flavors of hogweed, ketchup, lemon, salt as well as raw samples were collected from one of the reputable factories in Tehran and transferred to Tehran University of Medical Sciences for preparation. The necessary permission to take samples from the factory's products was obtained with a letter issued by the vice-chancellor of the university. All methods were performed in accordance with the university's research regulations.

### Sample preparation

Reflux extraction is one of the traditional methods for sample preparation that its efficiency has been confirmed^18^. The shell and kernel were separated. Then, The shell and kernel were powdered by an electric grinder. In the next step, 50 g of the sample was poured into a 500 ml round bottom Erlenmeyer flask. 100 ml of ethanolic KOH solution was added to the flask. Then, 0.5 ml of standard xylene as internal solution (at a concentration of 1 μg ml^−1^ in methanol) was added. This solution was refluxed for 3 h. Then, the prepared shell sample was filtered by porous filter No. 3 and the kernel sample was filtered by Buchner filter. In order to wash the sample, 20 ml of methanol and water in a ratio of 9:1 was added. Then, this solution with 50 ml of isooctane in a separatory funnel and shaken each time for 5 min, and collected the isooctane phase in a container and the resulting extract was washed with 100 ml of methanol and water in a ratio of 1:1. Isooctane solvent was extracted twice and each time with 50 ml in separatory funnel for 5 min. To the isooctane phase, 10 g of active sodium sulfate powder was added, shaken for 1 min by a shaker, then filtered through a sieve, and finally the isooctane phase was evaporated in rotary to a volume of 2 ml at 55° C.

### GC–MS analysis

In this study, the desired analytes were separated and identified by utilizing an Agilent 7890A gas chromatograph which possessed a 5975-mass selective detector (MSD, Agilent Technologies) and was equipped with Restek Rxi®-5MS fused-silica capillary column 5% Phenyl Methyl Silox (60 m × 0.32 mm i.d. and 0.25 µm film thickness). The flow of Helium (purity 99.999%) as a carrier gas was determined to be at the rate of 1.0 mL min^−1^.

The analysis situation in this research was conducted in the splitless mode and injector temperature of 290 °C. The temperature of the oven was firstly regulated at 70 °C for 2 min, accompanied by the temperature ramp of 20 °C min^−1^ to 220 °C. Then the oven temperature was raised to 295 °C at a rate of 5 °C min^−1^ for 8 min, and the solvent was retard for 4 min, to an all-run time of 34.5 min.

**Figure 1:**
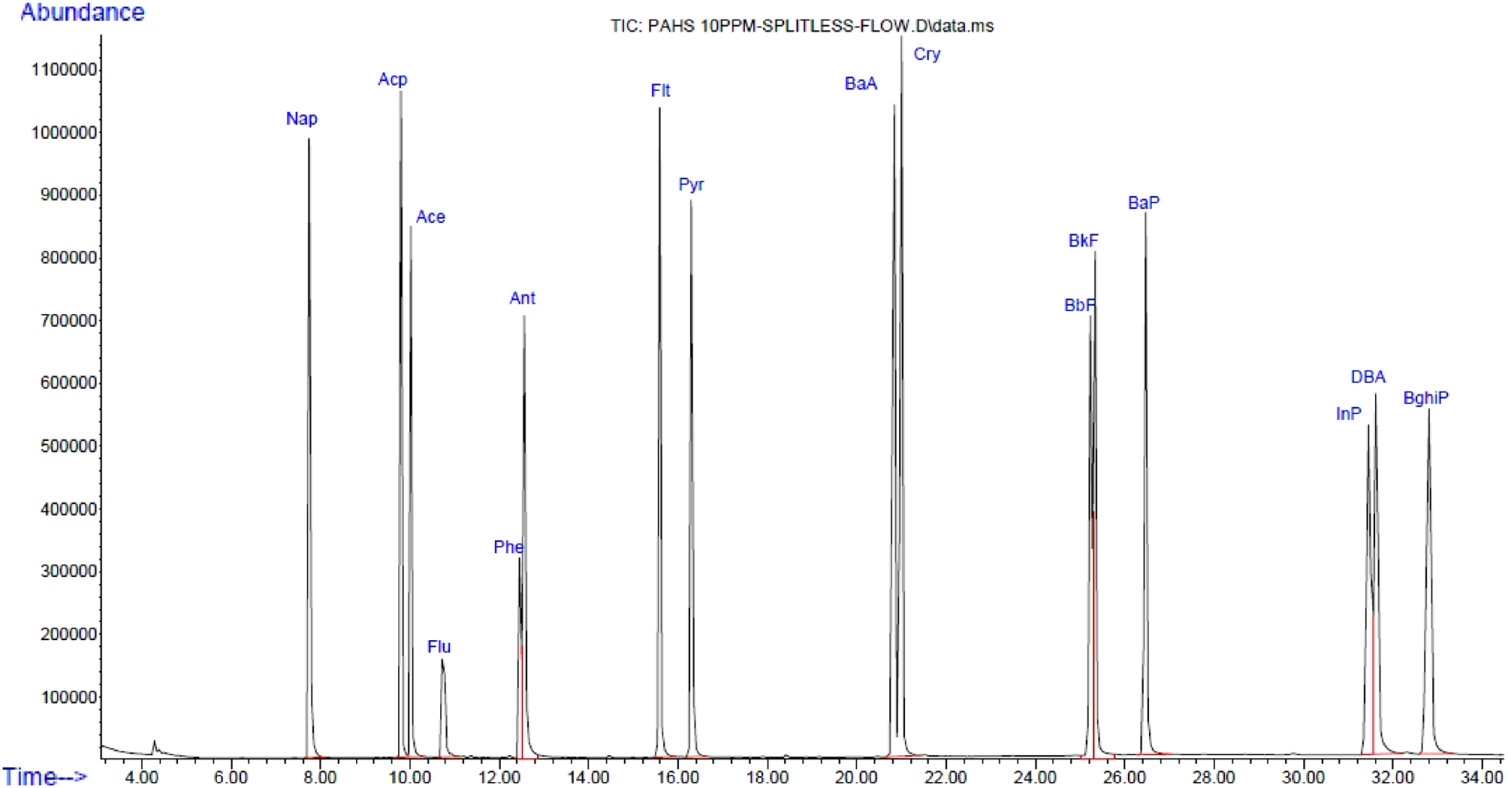
Chromatogram of standard mixture of 10 mg kg^−1^ polycyclic aromatic hydrocarbons (PAHs).

The MSD was worked in the mode of the electron impact (EI) (70 eV) by the ion source temperature at 230 °C. The MSD transfer line and temperature of the quadrupole were conducted to be 290 and 150 °C. Our research samples were extracted and injected at 2 µL into the GC–MS. The MSD system was generally planned in the selective ion monitoring (SIM) mode. The PAHs in the standard mixture were identified by comparing the chromatography based on the retention time (Fig. 1).

Extracted solution of Real samples and 5 point Calibration solutions were injected 3 times in the same sequence. Data acquisition were recorded and processed, for any of 16 PAHs, using the Agilent Qualitative and Quantitative analysis software. Target compounds were isolated using selective Ion monitoring (SIM) and identified by the combination of retention time and mass spectral match against the calibration standards measured simultaneously with the samples.The results were shown in the Table 2, after calculating the pre-concentration coefficient.

### Analytical performance

The Limit of detections (LODs) of method for each PAHs were obtained in the range of 3 to 4 µg kg^−1^ practically based on signal-to-noise ratio of 3. Limit of quantifications (LOQs) was 9–10 µg kg^−1^. For each PAHs, LOD and LOQ are also displayed in Table 1. Linear dynamic ranges (LDRs) were 10 to 400 µg kg^−1^ with coefficient of determination (R^2^) from 0.9898 to 0.9996. Quantification and identification ions (m/z) of 16 PAHs were shown at Table 1. Confirmation of the PAHs was established by the retention time and the presence of the target ions. The target ion abundances were determined by injection of PAHs standard under the same chromatographic condition but utilizing full-scan conditions with the mass/charge scan ranging from 40 to 550 m/z.

**Table 1:** Name of PAHs component, LOD, LOQ and Instrumental analyses characteristics.

No	PAH	Abrev	RT (min)	M/Z	LOD (µg kg^−1^)	LOQ (µg kg^−1^)
1	Naphthalene	Nap	7.742	128, 129, 102	3	9
2	Acenaphthylene	Acp	9.799	152,153,151	3	9
3	Acenaphthene	Ace	10.015	153, 154, 76	3	9
4	Fluorene	Flu	10.773	166, 165, 82	3	9
5	Phenanthrene	Phe	12.451	178, 176, 76	3	9
6	Anthracene	Ant	12.559	178, 179, 79	4	10
7	Fluoranthene	Flt	15.590	202, 203, 101	3	9
8	Pyrene	Pyr	16.294	202, 203, 200	3	9
9	Benz[a]anthracene	BaA	20.840	228, 226, 114	3	9
10	Chrysene	Cry	21.003	228, 226, 229	3	9
11	Benzo(b)fluoranthene	BbF	25.225	252, 253, 126	3	9
12	Benzo[k]fluoranthene	BkF	25.333	252, 253, 113	3	9
13	Benzo[a]Pyrene	BaP	26.470	252, 253, 250	3	9
14	Indeno[1,2,3-cd]pyrene	InP	31.449	276, 277, 138	4	10
15	Dibenzo[a,h]anthracene	DBA	31.611	276, 277, 139	4	10
16	Benzo[ghi]perylene	BghiP	32.802	276, 277, 138	4	10

RT, retention times of GC–MS chromatogram.

M/Z, selected ions for target pesticides used as quantifier and qualifier respectively.

### Statistical analysis

Mean and standard deviation were calculated by SPSS software. Kolmogorov–Smirnov test was used for parametric and non-parametric tests. All data were non-parametric. Therefore, Kruskal–Wallis and Mann–Whitney tests were used for significant differences between sunflower sunflower shells and kernels together.

## Results

The amounts of total PAHs in the shell and kernel of sunflower seeds with different flavors of ketchup, Hogweed (golpar), lemon, and salt and in raw samples were compared with each other. The mean and standard deviation of these PAHs are shown in Table 2. The PAHs of sunflower seeds were in the range of 0.4–3.2 mg kg^−1^. The lowest amount was related to hogweed kernel and the highest amount was related to lemon shell (Table 2). A significant difference was observed between amount of total PAHs of hogweed kernel and other kernels (*P* < 0.05). Furthermore, a significant difference was observed between lemon shell and lemon kernel (*P* = 0.02). A significant difference was also seen between the total PAHs concentrations in the roasted shell and roasted kernel of the sunflower seeds. The large amount in the shell is probably due to the direct contact of the shell with heat^14^. Also the raw samples did not have PAHs (Table 2).

**Table 2:** Average concentrations of total PAHs (mg kg^−1^) in the shell and kernel of roasted and raw sunflower seeds.

Samples	Average and standard deviation
Raw kernel	ND
Raw shell	ND
Hogweed kernel	0.4 ± 0
Hogweed shell	2.2 ± 1
Lemon kernels	0.68 ± 0.12
Lemon shell	3.2 ± 0.4
Ketchup kernel	1.8 ± 0.2
Ketchup shell	1.48 ± 1.4
Salt kernel	1.2 ± 0.8
Salt shell	2 ± 1.2

Low molecular weight PAHs were also measured, their average is shown in Table 3. Kernel and shell were compared for each type of flavoring. A significant difference was observed between lemon kernel and lemon shell (*p* < 0.05) and hogweed kernel and hogweed shell (*p* = 0.04). Furthermore, the shell and the kernel of all flavorings were compared. A significant difference was observed between the shell of lemon and the shell of other flavorings. In addition, a significant difference was observed between ketchup kernel and other kernel (*p* < 0.05).

**Table 3:** Average concentrations of low molecular weight of PAHs (mg kg^−1^) in the shell and kernel of roasted and raw sunflower seeds.

samples	Salt shell	Salt kernel	Ketchup shell	Ketchup kernel	Lemon shell	Lemon kernels	hogweed shell	hogweed kernel	Raw shell	Raw kernel
Average of LMW*	1.8 ± 1	0.6 ± 0.5	1.28 ± .48	1.6 ± 0	2.8 ± 0.8	0.68 ± 0.12	2.2 ± 1	0.2 ± 0.18	ND	ND

*LMW Low molecular weight.

PAH levels included PAH8 (B[a]A, CHR, B[b]F, B[k]F, B[a]P, D[a,h]A, I[c,d]P, and B[g,h,i]P) was calculated for all samples and was ND. For high molecular weight PAH, only fluoranthene was observed, the rest were ND. It is important to note that the heavier compounds, in addition to being more stable, have a higher lipophilic character, a characteristic that facilitates their absorption by the body^19^.

## Discussion

This study showed the role of food additives on the amount of PAHs in sunflower seeds. The roasting process produces PAHs in sunflower seeds. Unsaturated fatty acids, that is present in sunflower seeds in large quantities, such as linoleic acid and linolenic acid, can easily generate cyclic monomers through polymerization, and finally form PAHs precursors containing benzene rings^20,21^. In this study, high molecular weight PAH such as benzo[a]pyrene and chrysene not detected in sunflower seeds. The process of roasting sunflower seeds at 100 °C was done for ten to fifteen minutes depending on the moisture content of the seeds. These kind of PAHs are usually detected in conditions that the roasting process is carried out at high temperatures^22^. The coffee roasting process takes place at a temperature of 180 to 260 °C. Heavy PAHs such as chrysene, pyrene, benz[a]anthracene, and anthracene are detected^23^. In another study that was conducted for coffee beans, it was observed that the pyrene and chrysene were formed at a temperature of 260°C^24^. Therefore, it is obvious that heavy molecules are not detected due to the temperature and roasting time in this study.

### PAHs levels in sunflower seed treated with flavors

In this study, the amount of total PAHs in sunflower seed kernels with hogweed flavor was significantly different from the amount of total PAHs in other sunflower seed kernels with lemon, ketchup and salt flavors (*p* < 0.05). Moreover, the amount of low molecular weight PAHs in hogweed kernel was lower than sunflower seed kernel with other flavorings (*p* < 0.05).

Plants are unique in their ability to produce extraordinary effects^25^.The hogweed plant has therapeutic and nutritional properties. In Europe, it is used to treat diarrhea. In Asian countries, it is used as a food additive, spices^26,27^. Hogweed contains phenolic compounds including phenolic compounds gallic acids, rutin, coumarin and quercetin. Phenolic compounds of this plant have strong antioxidant properties^28^. One of the best-known effects of coumarin is its antioxidant properties^27^. Natural antioxidant compounds are safe compounds^29^. The presence of antioxidants is effective in determining the amount of PAHs in heated or smoked products^30^. The mechanism of action is that antioxidants can act as radical scavengers and suppressants for PAH formation. Also, since lipid is an important precursor in the production of PAHs, antioxidants can be used to prevent lipid oxidation to reduce the formation of PAHs^31^. In a study, the effects of different antioxidants on the formation of PAH in meat samples were investigated. After determining the most effective concentration, individual antioxidants (BHT, BHA, α-tocopherol, EGCG and sesamol) were added to the meat. Total PAH in all meat samples decreased with added antioxidants^31^. The results presented by Wang et al. (2019) and Cordeiro et al. (2020) show that phenolic compounds inhibit PAHs in two ways: scavenging free radicals and preventing the formation of PAHs through more complex interactions such as binding to PAH intermediates, but this issue needs more research^15^. In addition to the flavorings mentioned, reported meat flavorings such as onion, paprika, red pepper, black pepper powder, garlic and ginger prevent the formation of PAHs which can be caused by their antioxidant activity^32^. But, in this study, there was no significant decrease in the amount of total PAHs in the ketchup samples. Furthermore, the amount of PAHs with low molecular weight of ketchup kernel was significantly different from sunflower seed kernel with lemon and hogweed flavors. The amount of PAHs with low molecular weight in ketchup kernels was higher than in lemon and hogweed kernels (*p* < 0.05). Lycopene is a powerful antioxidant found in ketchup^33^. It is a natural carotenoid that can reduce the toxicity of PAHs in the body of living organisms^34^. Therefore, it does not have the ability to reduce PAHs in food environments. Although extensive research is needed in this regard.

In this study, the level of total PAHs in the kernels of sunflower seed samples processed with lemon was also low (Table 2). Wongmaneepratip et al. (2017) in a study, found that a slight increase in the pH value can significantly increase the PAH content in grilled chicken^17,35^.The addition of lemon juice lowers the pH and possibly affects the rate of the PAH formation reaction. Additionally, the sulfur dioxide compound in lemon juice may help reduce PAH by inhibiting Maillard reactions^36,37^. During Maillard reactions, a reaction between an amino acid and a reducing sugar produces a Schiff base. This Schiff base itself is unstable and is easily oxidized and enters the production cycle of Amadori compounds. These compounds may undergo intricate reactions (pyrolysis and polymerization) to form PAHs such as pyrene and BaA^38,39^. In this way, reducing the Maillard reaction helps to lower the PAHs. Similarly, Farhadian et al.^37^ explained that the addition of acidic substances, rich in polyphenols, i.e. lemon juice or tamarind to marinade treatments, reduced PAH formation in grilled beef. On the other hand, in the article by Cordeiro et al.^40^, excessive reduction of pH did not prevent PAHs production. Perhaps the reason is that acidic marinades (acetic acid, citric acid, lactic acid, etc.) greatly affect the structure of cooked meat^41,42^ and acid treatments reduce water holding capacity and increase fat and protein oxidation in meat. All these microstructural changes may cause non-inhibition of PAH at very low pH.

In this study, salt also had a positive effect on reducing the amount of PAHs with low molecular weight in sunflower seed kernels (Table 3). It has been reported that PAH formation is related to water content, and PAH content was higher in moist meat models compared to dry samples^17^. Many previous studies have shown that sodium can cause changes in water content, pH, or fatty acid composition and reduces the Maillard reaction, which indirectly changes PAHs amount^17,43^. Similar to the results of this research, it was observed in previous studies that salts (including calcium chloride, magnesium chloride, potassium lactate and calcium lactate) can reduce the pH of meat products and prevent PAH production^17^.

### PAHs levels in raw sunflower seed samples

The results indicated that raw samples are free of PAHs. PAHs can enter plants through water, soil and atmosphere^44^. Therefore, the amount of PAHs in raw sunflower seeds usually indicates the amount of pollution in the air, soil and water. In this study, the absence of PAHs in the raw samples indicates the absence of contamination of the agricultural environment of sunflower seeds. In our previous studies, PAHs was not reported in raw sunflower seed samples^14^. The difference between raw samples and roasted samples is that roasted sunflower seed samples are contaminated with PAHs during the roasting process. Therefore, PAHs formation is strongly affected by the cooking method (frying, grilling, baking, and roasting)^45^. Furthermore, PAHs with high molecular weight are formed at high temperatures of cooking. Reducing the temperature is one of the most effective ways to reduce PAHs in food.

## Conclusion

The amount of PAHs in sunflower seeds can be affected by different flavors. In this study, the role of flavors on the PAHs in roasted sunflower seeds was investigated. Considerable values were observed in the reduction of PAHs by hogweed flavoring. Considering that the formation of PAHs during thermal processes is unavoidable, it can be suggested to the food industry that hogweed is effective in reducing these contaminats. Also, since the raw samples did not have PAHs, the roasting process can lead to contamination of the seeds, especially the seeds shell. In this study, the amount of total PAHs in all flavors was higher in the shell, except for the ketchup sample, the amount of PAHs in kernel was higher, which was not statistically significant. Furthermore, PAHs with high molecular weight were not observed in the samples of roasted sunflower seeds. Therefore, this product will probably not be considered a threat due to the risk of carcinogenesis.

## Data Availability

The datasets generated and analyzed during the current study available from the corresponding author on reasonable request.
